# The Neonatal Innate Immune Response to Sepsis: Checkpoint Proteins as Novel Mediators of This Response and as Possible Therapeutic/Diagnostic Levers

**DOI:** 10.3389/fimmu.2022.940930

**Published:** 2022-07-04

**Authors:** Emily Hensler, Habesha Petros, Chyna C. Gray, Chun-Shiang Chung, Alfred Ayala, Eleanor A. Fallon

**Affiliations:** ^1^ Division of Surgical Research, Department of Surgery, Rhode Island Hospital, Providence, RI, United States; ^2^ Graduate Program in Biotechnology, Brown University, Providence, RI, United States

**Keywords:** sepsis, neonate, checkpoint inhibitor, PD-1, PD-L1, VISTA, HVEM

## Abstract

Sepsis, a dysfunctional immune response to infection leading to life-threatening organ injury, represents a significant global health issue. Neonatal sepsis is disproportionately prevalent and has a cost burden of 2-3 times that of adult patients. Despite this, no widely accepted definition for neonatal sepsis or recommendations for management exist and those created for pediatric patients are significantly limited in their applicability to this unique population. This is in part due to neonates’ reliance on an innate immune response (which is developmentally more prominent in the neonate than the immature adaptive immune response) carried out by dysfunctional immune cells, including neutrophils, antigen-presenting cells such as macrophages/monocytes, dendritic cells, etc., natural killer cells, and innate lymphoid regulatory cell sub-sets like iNKT cells, γδ T-cells, etc.

Immune checkpoint inhibitors are a family of proteins with primarily suppressive/inhibitory effects on immune and tumor cells and allow for the maintenance of self-tolerance. During sepsis, these proteins are often upregulated and are thought to contribute to the long-term immunosuppression seen in adult patients. Several drugs targeting checkpoint inhibitors, including PD-1 and PD-L1, have been developed and approved for the treatment of various cancers, but no such therapeutics have been approved for the management of sepsis. In this review, we will comparatively discuss the role of several checkpoint inhibitor proteins, including PD-1, PD-L1, VISTA, and HVEM, in the immune response to sepsis in both adults and neonates, as well as posit how they may uniquely propagate their actions through the neonatal innate immune response. We will also consider the possibility of leveraging these proteins in the clinical setting as potential therapeutics/diagnostics that might aid in mitigating neonatal septic morbidity/mortality.

## Prevalence and Burden of Neonatal Sepsis

Neonates accounted for 47% of all mortalities in children under five years worldwide in 2020 (1). Sepsis, defined as a dysfunctional immune response to infection resulting in life-threatening organ injury (2), is the third leading cause of death in this group after prematurity or complications occurring during birth (3). Neonatal sepsis accounts for 13% of neonatal deaths and 42% of mortality in the first week of life (4). Globally, there are an estimated 2200 cases of neonatal sepsis per 100,000 live births with mortality ranging from 11 to 19% (5). Preterm infants are particularly susceptible, with premature neonates being 1000 times as likely as term counterparts to experience sepsis, as well as suffering from higher mortality and long-term morbidity (6).

In the United States, there is an overall mortality rate of 10% for septic infants, with a sharp increase up to 30% for those with any comorbidity (7). 36% of premature neonates born before 28 weeks gestation had at least one episode of bacteremia during their initial hospitalization with a mortality rate of up to 50% (6). Neonates in the US also have the highest rate of ICU admissions for sepsis of any age group and disproportionately high health care costs of 2-3 times those of adult septic patients, accounting for $1.1 billion in annual health care costs (7). Despite the high prevalence and significant health care burden of neonatal sepsis, reductions in mortality have lagged behind those seen in the overall pediatric population (4). In addition, the percentage of deaths in children under five years of age attributed to neonates has risen (1) showing that there is still significant room for improvement in the care of this vulnerable population.

## Neonatal vs Pediatric Sepsis

The rising incidence of sepsis, as well as its significant rates of mortality and long-term morbidity, led to the development of consensus definitions and recommendations for adult patients in 1991 (8) which have had multiple revisions based on updated evidence (2). Initial guidelines for pediatric patients were published in 2020 (9) and included 77 recommendations on the management of sepsis in children, 49 of which were classified as weak recommendations based on limited and low-quality evidence. While neonates born at 37 weeks gestation or later are intended to be covered by these guidelines, premature infants (a significant proportion of the neonatal population) and studies addressing concerns specific to neonates, such as those related to perinatal infections, were excluded. This somewhat limits the application of these guidelines to neonatal sepsis.

Several distinct differences between neonates and older children need to be considered in the diagnosis and management of sepsis. Neonates likely have exposure to different pathogens than older infants or other children due to intrauterine infections or vertical transmission during birth (10). Neonates are also affected differently by exposure to certain organisms, with those often dismissed as contaminants in older children or adults causing significant morbidity and mortality in the neonatal population. The long-term impact of neonatal sepsis must also be considered given the rapid development of the brain during this period. Studies have shown that patients who experience sepsis as neonates go on to have neurodevelopmental changes that persist even decades later (11–13).

Even amongst neonates there is considerable heterogeneity. These differences are due to varied gestational age of the population, timing of sepsis (early vs late), and source of the infection (10). Rapid changes in renal function (14) and the ability to metabolize medications (15) also occur in the first few weeks of life, causing variability in how individual patients may respond to a given treatment. Changes in the normal values for vital signs (16) and common laboratory tests (17) also occur in the first weeks to months of life and most clinical signs of sepsis in children and adults lack specificity in neonates (10). Even blood cultures are unreliable, with as few as 1% of cases having positive cultures due to the need for small samples and antenatal maternal antibiotic administration. All of these issues present significant challenges in forming widely-applicable definitions and recommendations for the diagnosis and management of neonatal sepsis.

## Animal Models of Neonatal Sepsis

While well-established models of sepsis in adult animals have vastly increased our understanding of the immune response to sepsis, the unique challenges posed by neonatal septic patients necessitate ongoing research using neonatal animals. Several models for neonatal sepsis have been developed to meet this need. Cecal ligation and puncture (CLP) is a commonly used model of intraabdominal polymicrobial sepsis in adult mice (18). This model has limitations in neonates, largely due to their small size and increased risk of cannibalization of the surgically manipulated neonates by the mothers (19). The cecal slurry (CS) model was developed to combat these issues. In this model, the cecal contents of adult mice are mixed with crystalloid fluid to create the slurry, which is then administered *via* IP injection to the study animals. The cecal slurry model induces bacteremia with mortality typically occurring between 12- and 72-hours following injection (20). This timeline is similar to that seen in septic human neonates (21). In addition to induction of sepsis *via* IP injection, pure bacteremia models utilizing IV injection of pathogens (22) as well as pneumonia models using intranasal administration (23) have been developed in rodents.

Models have also been developed to closely mimic necrotizing enterocolitis (NEC), a disease process most commonly seen in premature infants. These models typically involve gavage feeding of formula and either a single pathogen (24) or polymicrobial slurry (25, 26), often followed by induction of hypoxia to mimic ischemia-reperfusion (27). While there are several clear advantages to studying this disease process in rodents, including lower cost and larger litter size than other species, the inability to study premature animals limits the applicability of these models (20). Similar models have been developed using piglets (26, 28, 29) and non-human primates (30), but these species are more expensive and more difficult to care for. An additional limitation to these models of neonatal sepsis is the exclusion of antibiotic treatment and supportive care that would typically occur in human patients (20).

## Immunology of Neonatal Sepsis

Due to a limited exposure to environmental microbes, neonates rely on the innate immune system, which offers a rapid, short-term, and unspecified response to microorganisms (31). After a pathogen bypasses epithelial barriers, its pathogen associated molecular patterns (PAMPs) are detected by pathogen recognition receptors (PRRs), such as toll-like receptors (TLRs) (32, 33). Binding to TLRs stimulates a response through the release of cytokines, chemokines, complement proteins, and coagulation factors (34–36). While neonates and adults have similar expression levels of TLRs, the subsequent responses from PAMP-TLR binding differs (37). Septic neonates have decreased production of proinflammatory cytokines [TNF-α, IFN-γ, and IL-1β (38–40)], potentially due to decreased production of intracellular mediators of TLR signaling (41).

Several immune cells, including neutrophils, play a major role in the innate immune response (42). In rats, neonates have smaller baseline neutrophil reserves with higher risk of depletion of these reserves following sepsis as compared to adults (43). Additionally, neonatal neutrophils have lower expression of adhesion molecules (44, 45), which facilitate binding to the vascular endothelium, reducing neutrophil migration to the site of infection by 50% (45). Neonatal neutrophils also exhibit reduced deformability, which, when coupled with sepsis-induced hypotension, may lead to microvascular occlusion and successive organ dysfunction (46). Neonatal neutrophils also have impaired NET formation (47), reduced phagocytic capabilities (48), and decreased levels of bactericidal proteins (49). In addition, they are less efficient in responding to apoptotic stimuli (50, 51), which may prevent the resolution of inflammation and lead to excessive tissue damage (52). Overall, the combination of these changes in neutrophil functions may make neonates more susceptible to sepsis than adults.

Other key immune cells also demonstrate significant differences in the neonatal immune response. Neonates have low levels of antigen presenting cells (APCs), monocytes, and dendritic cells (53). While these cells have comparable cell surface TLR expression to adult cells (53), they express lower levels of costimulatory molecules (CD80, CD40) (54). Such factors contribute to a reduced ability of neonates to protect themselves from an infection. Another difference between adults and neonates is seen in mast cells, which release more histamine upon stimulation in neonates (55), potentially contributing to vasodilation and septic shock in this population.

In contrast to decreased numbers of other immune cells, neonates have larger populations of NK cells than adults (56). Neonatal NK cells have increased expression of inhibitory receptors (CD94/NKG2A), lower cytotoxic ability towards their targets, and decreased degranulation ability, when compared to adult NK cells (57). IFN-γ release by neonatal NK cells appears to differ based on type of *in vitro* stimulation, with some experiments showing increased IFN production (57) and others showing decreased levels (39) as compared to adults.

γδ-T cells, while lymphoid in lineage, are also innate immune cells that provide protection from microbial infection through the release of IFN-γ (58). γδ-T cells found in human cord blood have decreased cytotoxic capacity (59), decreased cytokine release (60), and lower levels of perforin and granzyme B effector molecules. Neonatal γδ-T cells are immature, reaching adult levels of maturity by 2 years of age (61), and have limited ability to respond to bacterial infection. In contrast, following infection with influenza, neonatal γδ-T cells rapidly produce IL-17A and contribute to improved survival as compared to neonatal mice lacking these cells (62).

iNKT-cells are adaptive immune cells with innate-like functions that have been shown to play a role in the neonatal immune response. While these cells do not appear to express TLRs as other innate immune cells do, they are activated by proinflammatory cytokines or lipid antigens and trigger further cytokine production and activation of other immune cell populations (63). In neonatal mice, loss of these cells leads to improved survival following sepsis as compared to WT neonates (64). iNKT-cells also migrate to the peritoneal cavity and are important for the mobilization of macrophages following sepsis in neonatal mice. These findings suggest that iNKT-cells play an important role in the neonatal response to sepsis.

## Role of Checkpoint Proteins in Sepsis

**Figure 1:**
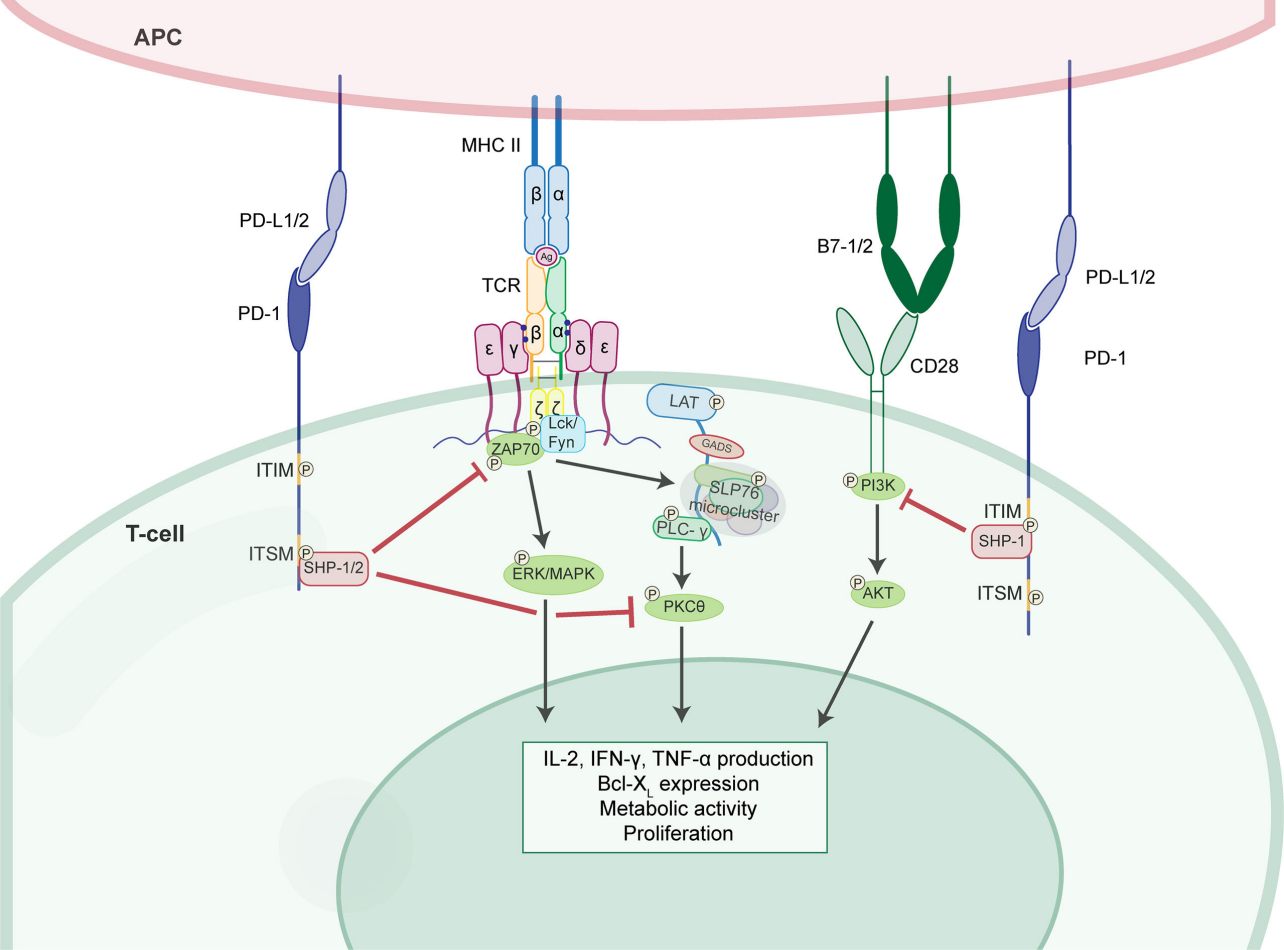
PD-1 suppresses T-cell activation by inhibiting several kinase pathways. T-cell activation occurs when the TCR binds the antigen (Ag) presented by MHC II. This first signal results in ZAP70, Lck, and Fyn recruitment to the CD3-ζ chain proximal to the TCR. The first signal promotes the ERK/MAPK and PKC-θ activation. The second signal occurs when CD28 binds to B7-1/2 and results in PI3K recruitment and downstream AKT pathway activation. PD-1 intrinsically suppresses T-cell activation. Upon interaction with PD-L1/2 the ITSM domain of PD-1 recruits SHP-1/2. Activated SHP-1/2 inhibit ZAP70 and PKC-θ phosphorylation. The ITIM domain of PD-1 is also phosphorylated and recruits SHP-1. This activated SHP-1 inhibits PI3K phosphorylation resulting in suppression of the AKT pathway. These mechanisms of PD-1 induced suppression results in reduced cytokine production, metabolic activity, proliferation, and B-cell lymphoma-extra large (Bcl-X_L_) mediated survival.

Two signals are required for T-cell activation to occur. First, an APC processes antigen and presents it to other cells *via* the MHC receptor (65). The T-cell receptor (TCR) binds to this antigen, creating the first signal. The second signal for activation is a co-stimulatory signal created by the binding of CD28 to B7, as an example (**Figure 1**). Concomitantly, co-inhibitory proteins, which can antagonize the second signal (66), are also present on various cell types, including APCs, T-cells, monocytes, macrophages, endothelial cells, tumor cells, etc. (67–70). These proteins allow for maintenance of self-tolerance in the body (71). The balance of co-stimulatory and co-inhibitory signals determines if the immune cell becomes activated or anergic/apoptotic (72).

During sepsis, the immune system generates simultaneous inflammatory and immunosuppressive responses (73), with balance of these responses necessary to prevent an overwhelming inflammatory response that could kill the host. While the inflammatory phase eventually peaks and returns toward baseline, many patients demonstrate profound long-term immunosuppression following sepsis (74). Immune checkpoint inhibitor proteins are often upregulated during the septic response and are thought to play a role in this immunosuppression (75). Here we will discuss the roles of several checkpoint inhibitors in sepsis and what is known about their involvement specifically in the neonatal immune response (**Table 1**).

**Table 1:** Brief summary of some selected checkpoint inhibitor ligands, expression, and signaling with relevance to the present discussion of neonatal sepsis.

Checkpoint Inhibitor	Ligands	Expression Pattern	Signaling Overview
PD-1	PD-L1 PD-L2	T-cells, NK cells, monocytes, dendritic cells (67), γδ-T cells (76)	SHP1/2 binds cytoplasmic tail -> inhibition of PI3K activation -> blocks Akt activation -> decreased Bcl-xL, IL-2, IFN-γ (77–79)
VISTA	VISTA VSIG3 PSGL-1	Spleen, thymus, bone marrow, leukocyte infiltrates, macrophages, monocytes, dendritic cells, T-cells, APCs (69)	SH2/SH3 binding -> STAT pathway activated -> inhibition of MAPK and NFκB pathways (80, 81)
HVEM	TNF LTα LIGHT BTLA CD160 HSV glycoprotein D	Fetal lung and kidney; adult spleen and peripheral blood leukocytes (70)	Recruitment of TRAF2 and TRAF5 -> transcription factor activation (eg NFκB and AP-1) (70)
BTLA	HVEM	Spleen, lymph nodes, CD4^+^ T-cells, B-cells, dendritic cells (82)	Recruitment of SHP1/2 -> dephosphorylation of PI3K (83)

### PD-1 (Programmed Cell Death Protein 1)

PD-1 is a well-studied checkpoint inhibitor protein expressed on activated T-cells, NK cells, monocytes, dendritic cells, and γδ-T cells (67, 76). It is composed of an extracellular domain, transmembrane domain, and cytoplasmic tail containing an immunoreceptor tyrosine-based inhibitory motif (ITIM) and an immunoreceptor tyrosine-based switch motif (ITSM) domain (84, 85). Upon binding of PD-1 to its ligand (PD-L1 or PD-L2), PD-1 is phosphorylated and the SHP1/2 complex is recruited to the cytoplasmic tail (77) (**Figure 1**). This leads to inhibition of CD28-mediated activation of PI3K, blocking downstream activation of Akt and leading to decreased Bcl-xL, IL-2, and IFN-γ production (78). PD-1 also inhibits phosphorylation of CD3ζ, and ZAP70, PKCθ (79), though these effects can be reversed *in vitro* by administration of IL-2, IL-7, or IL-15 (86). To exert these inhibitory effects, PD-1 must be in close proximity to the antigen receptor. To facilitate this, PD-1 has been shown to translocate to form micro clusters with T-cell receptors on the surface of T-cells (87).

Following the induction of experimental sepsis in adult mice, increased PD-1 expression can lead to exhaustion of T-cells, characterized by decreased proliferation as well as decreased production of IL-2, IFN-γ, TNF-α, and chemokines (88) with the greatest level of inhibition seen at low levels of TCR stimulation (67). T-cells also demonstrate a shift toward the regulatory phenotype following PD-1 activation (88). These changes are thought to contribute to the immunosuppression seen after sepsis. Similar changes have been documented in human samples as well, with splenic T-cells from adult septic patients showing decreased capacity for IFN-γ and TNF-α production (89). Expression levels of PD-1 and its ligands also change in septic patients, with increased PD-1 expression on CD4^+^ T-cells and increased PD-L1 expression on macrophages and endothelial cells.

PD-1^-/-^ adult mice develop autoimmune glomerulonephritis (90) and dilated cardiomyopathy (91), demonstrating the important role of this checkpoint inhibitor in maintenance of self-tolerance. However, in the response to sepsis, PD-1 activity leads to increased morbidity and mortality. PD-1^-/-^ adult mice have demonstrated improved survival following sepsis induced by CLP (92). Treatment of WT animals with anti-PD-1 or anti-PD-L1 antibodies similarly leads to improved survival in models of bacterial (93) and fungal (94) sepsis.

### PD-L1 (Programmed Death-Ligand 1)

PD-L1 is one of two ligands for PD-1. It is a type 1 glycoprotein containing IgC and IgV domains (84). The intracellular portion of the protein has been found to be highly conserved across species (67). In addition to its well-studied interaction with PD-1, PD-L1 has been found to interact with B7 (CD80), which is a known receptor for other immune checkpoint proteins such as CTLA-4 and CD28 (95). When bound to B7, PD-L1 inhibits T-cell activation independent of PD-1 involvement.

PD-L1 is widely expressed on both immune cells (including T-cells, B-cells, monocytes, macrophages, dendritic cells, bone marrow-derived mast cells, neutrophils, mesenchymal stem cells) (67, 68) and in various tissues, such as the cardiac endothelium, placenta, pancreatic islets, liver, lung, and skin (68). Its expression pattern varies based on the activation state of the cell, with lower baseline expression on T-cells and macrophages and significant upregulation on activated cells, though no such upregulation is noted on activated B-cells (67). It has also been found to be overexpressed on various cancers. This pattern of expression is thought to allow for regulation of the peripheral immune response and maintenance of self-tolerance in biologically vital tissues, as well as a mechanism for tumors to evade immune control.

In mouse models, PD-L1 deficiency exacerbates autoimmune diabetes (96) and increases susceptibility to experimental autoimmune encephalomyelitis (EAE) (97), suggesting a role in the prevention of autoimmune conditions. In sepsis, PD-L1 expression is upregulated on monocytes, dendritic cells, and capillary endothelial cells in the spleen (88). Monocyte PD-L1 expression levels also correlate with severity of illness and mortality in adult septic patients (98), suggesting a potential role as a prognostic marker in this population.

### VISTA (V-Domain Ig Suppressor of T-Cell Activation)

VISTA (PD-1H, VSIR, B7-H5, SISP1, Dies 1) (69, 99–101) is a relatively recent addition to the B7 family of checkpoint inhibitor proteins. It was identified *via* comparison of cell surface protein expression of resting vs activated T-regs and shares approximately 24% homology to PD-L1 (69). It is a type 1 transmembrane protein with an extracellular Ig-V domain, transmembrane segment, and cytoplasmic tail. It contains 2 invariant cysteine residues common to other B7 family members, plus an additional 4 cysteine residues that are unique to VISTA. These additional residues are highly preserved across species and are thought to allow for VISTA-VISTA interactions. In addition to self-binding, VISTA interacts with VSIG3, which is overexpressed on various GI cancers (102), and PSGL-1, an adhesion molecule involved in leukocyte rolling that is upregulated in inflammatory states (103).

VISTA’s cytoplasmic tail contains SH2 and SH3 binding domains, allowing for signaling *via* STAT proteins (80). This leads to downstream inhibition of TLR-mediated activation of MAPKs and the NFκB pathway *via* a reduction in TRAF6 (81). Overall, VISTA activity has an inhibitory effect on naïve and memory T-cell proliferation without inducing apoptosis in these populations (69). It also leads to decreased production of IL-2 and IFN-γ.

VISTA is primarily expressed in hematopoietic tissues, including the spleen, thymus, and bone marrow, as well as in tissues with significant leukocyte infiltrates such as the lung (69). It has a lower level of baseline expression in the heart, kidney, brain, and ovary. VISTA expression is highly upregulated on APCs during the inflammatory response and is also constitutively expressed on macrophages, monocytes, dendritic cells, and T-cells. Like PD-L1, VISTA deficiency has been found to exacerbate autoimmune conditions in mouse models, including EAE (104) and lupus (105). In addition, while many of these checkpoint inhibitor proteins have similar downstream effects on the immune response, VISTA and PD-1 have synergistic, non-redundant functions (106). This suggests that targeting a combination of these proteins may prove to be more beneficial than targeting them in isolation.

Adult VISTA^-/-^ mice have significantly decreased survival following septic insult as compared to their WT counterparts (107). They also demonstrate elevated serum markers of end-organ damage in the liver, as well as higher serum levels of several cytokines (IL-6, IL-10, TNF-α, MCP-1, IL-17F and IL-23). VISTA also appears to play an important role in the T-reg response to sepsis. While WT adult mice have increased T-reg abundance after sepsis, no such change is seen in VISTA^-/-^ mice. In addition, the survival of VISTA^-/-^ mice following sepsis returns to the WT baseline if VISTA-expressing T-regs are given *via* adoptive transfer prior to septic insult.

### HVEM (Herpesvirus Entry Mediator)

HVEM is a type 1 transmembrane receptor protein (108) containing 4 cysteine-rich domains that allow for ligand binding (70). Its cytoplasmic domain recruits TRAF2 and TRAF5, leading to downstream activation of various transcription factors, including NFκB and AP-1. HVEM is widely expressed throughout the body. High expression levels have been found at baseline in fetal lung and kidney, as well as adult spleen and peripheral blood leukocytes, with lower baseline expression in adult non-lymphoid tissues. HVEM is highly promiscuous in its interactions and can act as either ligand or receptor depending on its binding partner. It has several known ligands, including TNF, LTα, LIGHT, BTLA, CD160, and HSV glycoprotein D (83, 108–110).

LIGHT is a type II transmembrane protein (109) that is expressed in the spleen and lymph nodes, as well as on macrophages, T-cells, and immature dendritic cells (111). Its expression is inducible and its binding with HVEM stimulates proliferation of T-cells, induces IFN-γ production, and weakly stimulates NFκB-driven transcription (109). It also blocks proliferation of tumor cells *in vitro*. LIGHT also interacts with LTβR (112). This interaction produces a wide range of downstream effects, including cell apoptosis, lipid metabolism, and regulation of lymph node formation.

BTLA is a member of the Ig superfamily of proteins and contains 2 ITIM domains (113). BTLA is expressed in the spleen and lymph nodes, with low-level baseline expression on CD4^+^ T-cells that significantly increases following T-cell activation (82). Its interaction with HVEM is unique in that it acts as a bidirectional switch, with opposing downstream effects depending on which protein acts as receptor and which as ligand, as well as the membrane conformation of the involved proteins (83, 114).

In adult mice after CLP, increased BTLA and HVEM-expressing macrophages, monocytes, dendritic cells, and neutrophils have been found in the peritoneal cavity (115), suggesting a role for these checkpoint proteins in local response to infection. BTLA knockout animals have increased survival, decreased indices of organ injury, and reduced peritoneal bacterial burden following CLP (115), while those treated with an agonistic BTLA antibody show increases in cytokine production, recruitment of inflammatory cells to the peritoneal cavity, and mortality (116).

Higher levels of soluble BTLA have also been found in septic patients, and these levels appear to correlate with severity of disease (117). BTLA expression on CD4^+^ T-cells also correlates with severity of sepsis in ICU patients and is associated with increased risk of developing nosocomial infections (118). The immunosuppressed phenotype seen in these patients with higher BTLA expression may in part be explained by the increased apoptosis of T-cells seen following BTLA activation. These findings suggest a role for BTLA in prognostication and management of septic patients.

## Checkpoint Proteins in Neonates

While the role of checkpoint inhibitor proteins has been extensively studied in adults, significantly less data exists in neonates. In mice, PD-1^-/-^ neonates have been found to have a significant survival benefit following CS-induced sepsis as compared to WT neonates (119), mirroring the survival benefit seen in adult knockouts after CLP. These PD-1 knockouts also have increased production of cytokines following sepsis, specifically IL-6, IL-10, and TNF-α, as well as differences in the cell composition of peritoneal infiltrates. PD-1 also appears to play a role in modulating the response of neonatal iNKT-cells to sepsis, with both PD-1^-/-^ and iNKT-cell^-/-^ neonates showing similar effects on peritoneal macrophage populations that are distinct from those seen in WT neonates following CS (64).

PD-1 expression on monocytes has been studied in premature human infants, who have been found to have a lower baseline expression than their term counterparts (120). Premature infants with sepsis, however, have a significantly higher percentage of PD-1-expressing monocytes, with even higher expression levels seen in those who died of septic shock. PD-1 expression has also been studied in the context of inflammation due to biliary atresia. Infants diagnosed with biliary atresia have been found to have increased PD-1 expression on hepatic and circulating T-cells, as well as lower levels of IFN-γ in the liver (121). In a virus-induced biliary atresia model in mice, PD-1 blockade has been shown to lead to increased levels of AST, ALT, and IFN-γ, suggesting that PD-1 plays a role in mitigating liver injury in this disease process.

In humans, stimulation of CD4^+^ T-cells isolated from neonates with *Staphylococcus aureus* leads to a conversion of those cells to FOXP3^+^ regulatory T-cells (122). Blocking PD-L1 prevents this shift from occurring, suggesting that PD-L1 plays a role in controlling the immune response. BTLA expression has also been studied in human neonates, with higher levels of expression on dendritic cells of septic vs nonseptic patients (123). Samples from septic neonates also showed decreased T-cell proliferation and decreased levels of maturation markers on BTLA^+^ dendritic cells. The higher level of BTLA expression also correlated with decreased phagocytosis and bactericidal ability, as well as with the severity of sepsis in these patients.

Overall, these findings suggest that while there are significant differences in the neonatal immune response as compared to adults, checkpoint inhibitor proteins play an important role in the immune responses of both populations. These findings also suggest that checkpoint inhibitors play some of their most significant roles in ‘innate’ as opposed to simply ‘adaptive’ immune responses to sepsis. Other innate immune cells, such as neutrophils and γδ-T cells, are likely also affected by loss or blockade of checkpoint inhibition, but further research is needed to explore the effects of manipulating various checkpoint inhibitors on these cells. It is clear from the paucity of data in this specific and vulnerable population that much work remains to be done to understand the mechanisms by which checkpoint inhibitor proteins impact the neonatal immune response before they can be studied as potential therapeutic targets.

## Clinical Applications

While the roles of various checkpoint inhibitor proteins have been extensively studied in the setting of sepsis, their use as therapeutic targets in human patients is just beginning to be explored. Several phase 1b clinical trials have been undertaken to look at the safety of several compounds that target PD-1 and/or PD-L1 in septic patients (124, 125). These trials have not revealed any increase in cytokine levels or significant safety concerns, though they only include a small number of patients. Expanded trials will be necessary to quantify the risk of autoimmune side effects that could result from loss of checkpoint inhibition leading to impaired self-tolerance. These side effects have the potential to be especially concerning in the neonatal population as these patients have an underdeveloped immune system and are somewhat fragile as compared to adults. No trials have been performed using VISTA, HVEM, or any of their ligands as therapeutic targets and none have been performed in neonates. These provide areas of opportunity for further study and drug development.

**Figure 2:**
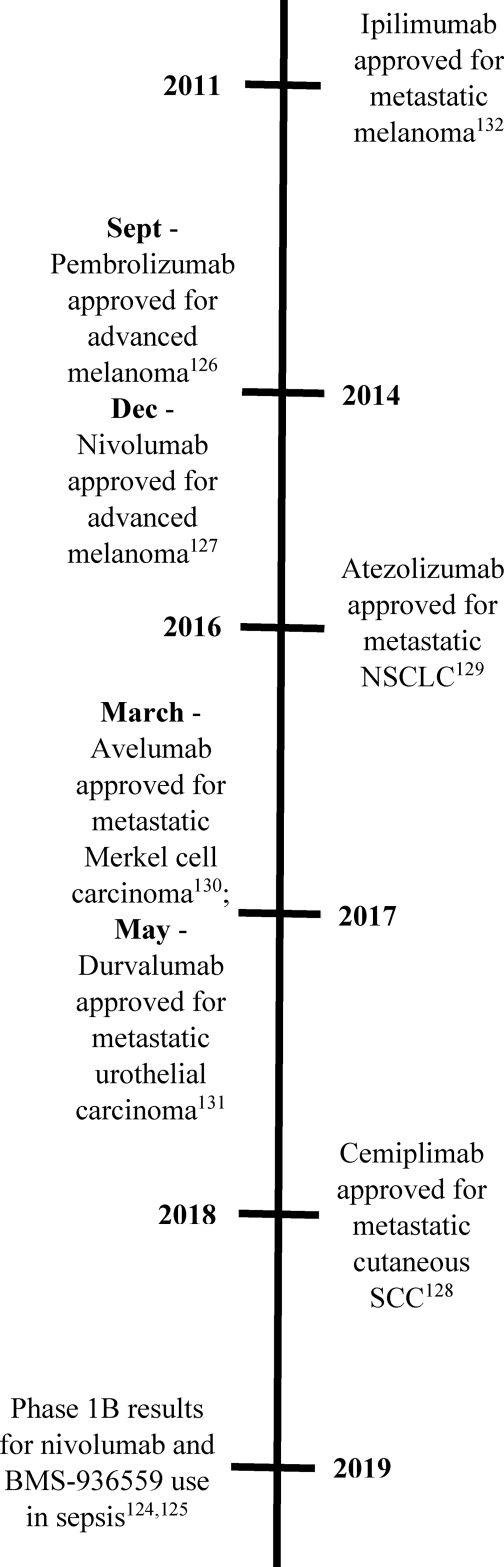
Timeline of therapeutic applications of checkpoint inhibitor proteins. This timeline includes the initial FDA approval for each of the approved medications targeting checkpoint inhibitor proteins (PD-1, PD-L1, and CTLA-4), as well as the publications of phase 1 clinical trial results for two of these medications in septic patients.

Checkpoint inhibitor proteins are well-established therapeutic targets in oncology (**Figure 2**). Three medications have been developed and approved to target PD-1 [pembrolizumab (126), nivolumab (127), cemiplimab (128)], three for PD-L1 [atezolizumab (129), avelumab (130), durvalumab (131)], and one for CTLA-4 [ipilimumab (132)]. These medications are used to treat a wide variety of cancers, including breast, lymphoma, skin, lung, and GI tumors. Unfortunately, not all tumors respond to these medications due to different levels of expression of the checkpoint proteins (133). Combinatorial therapies have also been studied with success in reducing death and/or disease progression in several trials (134). While these medications have been used successfully in the setting of cancer immunotherapy, further trials will need to be done in septic patients to determine if the positive results seen in a chronic process such as cancer will also be demonstrated in the more acute setting of sepsis.

## Conclusions

Despite advances in knowledge about the mechanisms, diagnosis, and management of sepsis, significant gaps remain in the setting of neonatal sepsis, which continues to be a large burden on healthcare globally. The differences between the neonatal and adult immune response make it impossible to extrapolate findings in adult animals or patients to neonates, and further work needs to be done to understand how sepsis affects this population. Checkpoint inhibitor proteins, such as PD-1, PD-L1, VISTA, and HVEM, have been shown to play an important role in modulating the immune response to sepsis in adults. Significantly less data exists for neonates, providing an additional area for further research. Current data suggests that these proteins may prove to be useful for diagnosis, prognostication, and even treatment of septic patients, but there is still more work to be done before this can be applied in clinical practice.

## Author Contributions

EH conducted the majority of the literature search and writing of the manuscript, and also contributed to the preparation of the figures. HP contributed to the literature search and writing of the manuscript. C-SC provided revisions and conceptual feedback. CG contributed to the preparation of the figures and provided revisions and conceptual feedback. EF and AA, who contributed equally as senior authors to this work, provided the initial framework for the review and contributed substantial revisions and conceptual feedback for the manuscript and figures. All authors reviewed and approved the final manuscript.

## Funding

This work was supported by the National Institutes of Health [R35 GM118097 (AA, C-SC), R25 GM083270 (CG), T32-HL134625 (CG) and T32 GM065085 (EH)].

## Conflict of Interest

The authors declare that the research was conducted in the absence of any commercial or financial relationships that could be construed as a potential conflict of interest.

## Publisher’s Note

All claims expressed in this article are solely those of the authors and do not necessarily represent those of their affiliated organizations, or those of the publisher, the editors and the reviewers. Any product that may be evaluated in this article, or claim that may be made by its manufacturer, is not guaranteed or endorsed by the publisher.

## Abbreviations

(-/-), Knockout; Akt, Protein kinase B; ALT, Alanine transaminase; AP-1, Activator protein 1; APC, Antigen presenting cell; AST, Aspartate aminotransferase; Bcl-xL, B-cell lymphoma- extra large; BTLA, B and T lymphocyte attenuator; CD, Cluster of differentiation; CLP, Cecal ligation and puncture; CS, Cecal slurry; CTLA, Cytotoxic T-lymphocyte associated protein; Dies1, Differentiation of embryonic stem cells 1; EAE, Experimental autoimmune encephalomyelitis; FOXP3, Forkhead box P3; GI – Gastrointestinal; HSV, Herpes simplex virus; HVEM, Herpesvirus entry mediator; ICU, Intensive care unit; IFN-γ, Interferon gamma; IgC, Immunoglobulin constant; IgV, Immunoglobulin variable; IL, Interleukin; iNKT, Invariant natural killer T-cell; IP, Intraperitoneal; ITIM, Immunoreceptor tyrosine-based inhibitory motif; ITSM, Immunoreceptor tyrosine-based switch motif; IV, Intravenous; LIGHT, Homologous to lymphotoxin, exhibits inducible expression and competes with HSV glycoprotein D for binding to HVEM, a receptor expressed on T-lymphocytes; LTα, Lymphotoxin alpha; LTβR, Lymphotoxin beta receptor; MAPK, Mitogen-activated protein kinase; MCP, Monocyte chemoattractant protein; MHC, Major histocompatibility complex; NEC, Necrotizing enterocolitis; NET, Neutrophil extracellular trap; NFκB, Nuclear factor kappa light chain enhancer of activated B-cells; NK, Natural killer; PAMPs, Pathogen-associated molecular patterns; PD-1, Programmed cell death protein 1; PD-1H, Programmed death 1 homolog; PD-L1, Programmed death ligand 1; PD-L2, Programmed death ligand 2; PI3K, Phosphatidylinositol 3-kinase; PKCθ, Protein kinase C theta; PRRs, Pathogen recognition receptors; PSGL-1, P-selectin glycoprotein ligand 1; SHP, Src homology 2 domain-containing protein tyrosine phosphatase; SISP1, Stress induced secreted protein 1; STAT, Signal transducer and activator of transcription; TCR, T-cell receptor; TLR, Toll-like receptor; TNF-α, Tumor necrosis factor alpha; TRAF, Tumor necrosis factor receptor-associated factors; T-reg, Regulatory T-cell; VISTA, V-domain Ig suppressor of T-cell activation; VSIG3, V-set and immunoglobulin domain containing 3; VSIR, V-set immunoregulatory receptor; WT, Wildtype; ZAP70, Zeta-chain-associated protein kinase 70.
